# Anti-saccade error rates are associated with somatic depressive symptoms in cocaine use disorder

**DOI:** 10.1177/02698811251399579

**Published:** 2025-12-26

**Authors:** Constanza de Dios, Heather E. Webber, Margaret C. Wardle, Jin H. Yoon, Michelle A. Patriquin, Jessica N. Vincent, Joy M. Schmitz, Scott D. Lane

**Affiliations:** 1 Faillace Department of Psychiatry and Behavioral Sciences, McGovern Medical School, University of Texas Health Science Center at Houston, Houston, TX, USA; 2 Department of Psychology, University of Illinois at Chicago, Chicago, IL, USA

**Keywords:** cocaine use disorder, anti-saccade, inhibitory control, depression, sleep

## Abstract

**Background:**

Inhibitory control deficits are associated with cocaine use disorder (CUD) development and maintenance. Additionally, drug use and inhibitory control can be negatively affected by depressive symptoms, including somatic factors like sleep disturbance and fatigue.

**Aim:**

The current study assessed the relationship between inhibitory control and depressive symptoms among individuals initiating CUD treatment. We examined associations among anti-saccade response inhibition performance, total scores on the Beck Depression Inventory-II (BDI-II), and individual BDI-II items.

**Methods:**

*N* = 101 patients enrolled in a clinical trial for CUD completed drug-specific anti-saccade and depression (BDI-II) measures prior to treatment. Generalized linear models tested the associations of anti-saccade error rate with stimulus type (cocaine, neutral) and with BDI-II total score. Penalized regression then modeled the error rate among the entire set of BDI-II items to select the most relevant symptom correlates.

**Results:**

Anti-saccade error rates were higher on cocaine relative to neutral trials (*p* < 0.001), confirming attentional bias. Error rates were positively associated with BDI-II total scores, controlling for demographic and recent cocaine use variables (*p* < 0.001); this association did not differ by stimulus content (*p* = 0.742). Among BDI-II items, Loss of Pleasure, Crying, Agitation, Changes in Sleeping Pattern, Concentration Difficulty, Tiredness, and Loss of Interest in Sex were retained by the penalized regression of error rates.

**Conclusions:**

Attentional bias was drug-specific, and overall error rates were strongly related to somatic factors underlying depression in CUD. The association between inhibitory control and depression in CUD may be driven by physiological symptomatology, including sleep impairment and fatigue.

**ClinicalTrials.gov:**

NCT02896712.

## Introduction

Executive dysfunction is related to the development, maintenance, and relapse of substance use disorder (SUD; Jarmolowicz et al., 2013), prompting examination of mechanisms of cognitive enhancement as potential targets for SUD treatment (Aharonovich et al., 2018; Rezapour et al., 2016). Inhibitory control, one of the components of executive function (Diamond, 2013; Miyake et al., 2000), refers to the ability to suppress prepotent responses in order to select relevant information while resisting interference from irrelevant information (Ainsworth and Garner, 2013). There is high interest in SUD applications of laboratory-based assays of inhibitory control as it plays a key role in drug cue reactivity, craving, and relapse (Marhe et al., 2013; Roberts et al., 2014).

Laboratory-based behavioral measures of inhibitory control include tasks that capture prepotent response inhibition (Friedman and Miyake, 2004; Hallett, 1978). For example, in an anti-saccade task (Hallett, 1978), individuals are instructed to direct their eye movement (saccade) to the opposite direction of a stimulus presented in the peripheral visual field. In order to execute a correct anti-saccade, the individual must be able to detect the stimulus in the peripheral visual field, and crucially, exercise response inhibition to suppress reflexive saccades toward the salient peripheral visual stimulus and produce a voluntary saccade in the opposite location. Clinical populations (e.g., SUD, mood disorders, schizophrenia) show poorer response inhibition on the anti-saccade task as evidenced by overall higher error rates compared to healthy controls (Ainsworth and Garner, 2013; Clementz et al., 1994; Sereno and Holzman, 1995). In the addiction context, variants of this task also indicate attentional bias, that is, inhibitory control deficits that are specific to addiction-relevant stimuli. Attentional bias in clinical populations is captured by presenting clinically salient images (e.g., drug cues) and neutral images for a portion of the trials, which leads to differential accuracy (more errors) on the salient versus neutral trials (Anderson et al., 2011). These inhibitory control difficulties are related to subjective experiences in addiction, such as craving (Dias et al., 2015), and to outcomes in treatment (de Dios et al., 2021), suggesting the need to understand the origins and correlates of these inhibitory deficits.

In SUDs, investigations of the anti-saccade response inhibition have mostly been in nicotine, alcohol, and cannabis use (O’Neill et al., 2020; Roberts et al., 2014; Schröder and Mühlberger, 2022; Yoon et al., 2019). Recent work has focused on psychostimulant use including cocaine use disorder (CUD). People with CUD show lower accuracy on drug-related relative to neutral trials (Dias et al., 2015; Tannous et al., 2019), and responses on this task are related to experiences of obsessive/compulsive use that feels outside conscious control (Dias et al., 2015), and to outcomes in CUD treatment (de Dios et al., 2021). However, the sources and correlates of response inhibition difficulties in CUD are unclear, making them more difficult to address in treatment.

One potential contributor to response inhibition difficulties in CUD is co-morbid depressive symptoms. Response inhibition and attentional bias as measured on the anti-saccade task are well-established in mood and anxiety disorders, with higher error rates observed in individuals with major depression (Ainsworth and Garner, 2013; Grahek et al., 2018). Depressive symptoms are common in CUD (Conway et al., 2006) and are a poor prognostic sign in CUD treatment (Palazón-Llecha et al., 2024). Furthermore, CUD populations may exhibit specific depression symptomatology that leans toward somatic qualities, based on prior factor analyses of the Beck Depression Inventory-II (BDI-II) in stimulant-using populations. Notably, the three-factor structure of the BDI-II, comprising distinct Cognitive, Affective, and Somatic dimensions, has shown better fit in cocaine- and methamphetamine-using populations compared to single- or two-factor solutions (Buckley et al., 2001; Johnson et al., 2006; Seignourel et al., 2008). Stimulant users in Johnson et al. showed stronger endorsements of the Somatic dimension than either Cognitive or Affective factors, which suggests somatic items of the BDI-II (i.e., Loss of Energy, Changes in Sleeping Pattern) might contribute most strongly to depressive symptoms in CUD.

Depression may particularly relate to response inhibition via sleep-related symptoms. Importantly, emerging evidence suggests that sleep may be a critical, yet underexplored, factor influencing both inhibitory control and depressive symptomatology. Rapid eye movement sleep is associated with increased activity in brain regions involved in saccadic control (Hong et al., 1995). Sleep deprivation has been shown to impair saccadic performance, reducing peak velocity and accuracy while increasing latency (Zils et al., 2005), and individuals with insomnia exhibit altered attentional patterns, including delayed vigilance and increased focus on fatigue-related facial cues (Akram et al., 2018; Woods et al., 2013). Furthermore, an emerging area of research posits tear fluid as a potential biomarker of multiple systemic and psychiatric conditions. A hypothesized relationship among sleep deprivation, ocular dryness, and depression symptoms has implicated inflammatory cytokines in tear fluid (Vavilina et al., 2023). These findings suggest that sleep disturbances may modulate eye movement behavior and attentional control.

Notably, sleep disturbances are highly prevalent in depression, with insomnia symptoms affecting the majority of individuals with major depressive disorder (Sunderajan et al., 2010). Rather than being a secondary symptom, insomnia may precede and exacerbate depressive episodes, and its persistence is associated with recurring depressive episodes. Critically, sleep disturbances are also highly prevalent in the CUD population (Webber et al., 2025) and are known to increase craving and drug-seeking after withdrawal (Chen et al., 2015). Indeed, sleep disturbances may operate mechanistically in CUD by increasing depression: in a prior mediational study, poor sleep was associated with worse treatment outcomes in CUD via mediating variables including depression and increased craving (Winhusen et al., 2019). These hypothesized relationships between depression, sleep, response inhibition, and CUD also present multiple potential points of intervention. However, the relationship between sleep disturbances, depressive symptoms, and inhibitory control in CUD is understudied—establishing these relationships is a key first step to potential interventions.

The current study examined anti-saccade performance in patients prior to undergoing treatment for CUD, and its association with sociodemographic, clinical, and depressive symptoms (BDI-II) assessed at baseline prior to initiating CUD treatment. We hypothesized that anti-saccade errors would be higher on cocaine compared to neutral trials based on documented attentional bias in CUD (Dias et al., 2015; Tannous et al., 2019). We also hypothesized that baseline BDI-II scores would be positively associated with anti-saccade errors, particularly on cocaine trials, and that BDI-II items reflecting somatic symptoms—especially those related to sleep and fatigue—would be most predictive of inhibitory control deficits.

## Method

### Participants and study design

The current study utilized data collected from the SMART trial for the treatment of CUD, (NCT02896712; Schmitz et al., 2024). Participants were treatment-seeking individuals who met current Diagnostic and Statistical Manual of Mental Disorders, fifth edition (DSM-5) criteria for CUD of at least moderate severity (⩾4 symptoms). The trial excluded individuals with medical conditions (e.g., severe cardiovascular disease, severe liver impairment) or taking medications (e.g., propranolol, phenytoin, warfarin, or diazepam) known to be contraindicated for modafinil pharmacotherapy. Medications reported by participants at baseline are summarized in Table S1. Following intake and baseline measures, CUD participants were randomized to treatment group. Full study details are published elsewhere (Schmitz et al., 2024). The study was conducted in accordance with the Declaration of Helsinki; all procedures were approved by the local UTHealth Houston IRB (HSC-MS-15-0595), and participants provided written informed consent.

After consenting and prior to treatment assignment, participants completed an intake evaluation process consisting of anti-saccade measures described below, along with the collection of demographic information and recent cocaine use (number of days in the past 30 days) as measured by the Addiction Severity Index-Lite (Cacciola et al., 2007).

Depressive symptoms were measured at baseline by the Beck Depression Inventory-II (BDI-II) (Beck et al., 1996). The BDI-II consists of 21 items assessing self-reported symptoms during the past 2 weeks on a 0–3 scale. Total scores range from 0 to 63. Example items and response options are shown in Table 1 (e.g., Tiredness: 0 = “I am no more tired or fatigued than usual,” 1 = “I get more tired or fatigued more easily than usual,” 2 = “I am too tired or fatigued to do a lot of the things I used to do,” 3 = “I am too tired or fatigued to do most of the things I used to do”). The BDI-II showed high internal reliability (Cronbach’s α = 0.941).

**Table 1.** Participant demographic and baseline characteristics.

Characteristic	*N* = 101
Demographic characteristics	
Age, *M* (SD)	49.5 (7.9)
Sex, *n* (%)	
Male	82 (81.2)
Female	19 (18.8)
Race, *n* (%)	
Black/African American	76 (75.2)
White	15 (14.9)
Asian	2 (2.0)
More than one race	4 (4.0)
Unknown/not reported	4 (4.0)
Ethnicity, *n* (%)	
Not Hispanic	89 (88.1)
Hispanic	12 (11.9)
Education years, *M* (SD)	12.9 (1.5)
Past 30 days cocaine use (days), *M* (SD)	17.2 (9.5)
BDI-II Total Score, *M* (SD)	15.3 (12.3)
BDI-II Categories, *n* (%)	
No depression (0–13)	55 (54.5)
Mild depression (14–19)	10 (9.9)
Moderate depression (20–28)	15 (14.9)
Severe depression (29–63)	21 (20.8)
BDI-II items retained by the LASSO regression	
Loss of Pleasure, *n* (%)	
I get as much pleasure as I ever did from the things I enjoy	37 (36.6)
I don’t enjoy things as much as I used to	41 (40.6)
I get very little pleasure from the things I used to enjoy	17 (16.8)
I can’t get any pleasure from the things I used to enjoy	6 (5.9)
Crying, *n* (%)	
I don’t cry anymore than I used to	63 (62.4)
I cry more than I used to	28 (27.7)
I cry over every little thing	2 (2.0)
I feel like crying, but I can’t	8 (7.9)
Agitation, *n* (%)	
I am no more restless or wound up than usual	56 (55.4)
I feel more restless or wound up than usual	31 (30.7)
I am so restless or agitated that it’s hard to stay still	12 (11.9)
I am so restless or agitated that I have to keep moving or doing something	2 (2.0)
Changes in Sleeping Pattern, *n* (%)	
I have not experienced any change in my sleeping pattern	37 (36.6)
I sleep somewhat *more* than usual OR I sleep somewhat *less* than usual	41 (40.6)
I sleep a lot *more* than usual OR I sleep a lot *less* than usual	11 (10.9)
I sleep most of the day OR I wake up 1–2 hour early and can’t get back to sleep	12 (11.9)
Concentration Difficulty, *n* (%)	
I can concentrate as well as ever	49 (48.5)
I can’t concentrate as well as usual	32 (31.7)
It’s hard to keep my mind on anything for very long	17 (16.8)
I find I can’t concentrate on anything	3 (3.0)
Tiredness/Fatigue, *n* (%)	
I am no more tired or fatigued than usual	42 (41.6)
I get more tired or fatigued more easily than usual	40 (39.6)
I am too tired or fatigued to do a lot of the things I used to do	15 (14.9)
I am too tired or fatigued to do most of the things I used to do	4 (4.0)
Loss of Interest in Sex, *n* (%)	
I have not noticed any recent changes in my interest in sex	60 (59.4)
I am less interested in sex than I used to be	27 (26.7)
I am much less interested in sex now	13 (12.9)
I have lost interest in sex completely	1 (1.0)

Strengthening the Reporting of Observational Studies in Epidemiology (STROBE) recommendations were used to ensure proper reporting of methods, results, and discussion in this cross-sectional study (Supplemental Materials).

### Anti-saccade task

Baseline eye-tracking testing consisted of blocks of trials in which participants were instructed to direct their eyes toward (pro-saccade) or away (anti-saccade) cocaine-specific or matched-neutral stimuli. Experimental procedure details have been published elsewhere (Dias et al., 2015) and are summarized as follows. Participants were tested with a 60-Hz infrared binocular eye-tracker. They performed four, 24-trial counterbalanced testing blocks (two pro-saccade, two anti-saccade). Each 24-trial block included 12 cocaine- and 12 neutral-stimulus trials, presented randomly but with no trial type occurring on more than three consecutive trials. Our research group has found reliable response latencies across studies based on these trial sizes (Table S2). Blocks were separated by a 60-second rest period. Trials were separated by an intertrial interval averaging 600 ms (with random jitter between 350 and 750 ms to prevent anticipatory effects). Cocaine-related and neutral images were matched as closely as possible for color and complexity. A schematic of the trial sequence and example images are available in Supplemental Figures S3 and S4.

### Data analytic strategy

Anti-saccade trial accuracy and response times were obtained from the raw data. Response time was operationally defined as the latency to execute a saccade, where the position of the eyes exited the defined space around a centered on-screen fixation cross and entered the defined stimulus space, that is a rectangular on-screen space occupied by the picture (error) or directly opposite and the same size as the picture (correct anti-saccade). A correct anti-saccade was defined as an eye movement away from on-screen picture. Based on the anti-saccade literature and suggested best practices, trials with latencies <90 ms were considered anticipatory and excluded from analysis (Abel and Douglas, 2007; Tannous et al., 2019).

Data analyses occurred in three stages. First, to confirm an attentional bias effect, anti-saccade error rates were cast into a generalized linear mixed model (GLMM), testing the outcome as a function of trial type (cocaine, neutral), including subject as random effects. Second, to explore baseline predictors of anti-saccade error rates, error rates averaged across trial types were cast in a multiple regression on a set of demographics (age, sex, race, ethnicity, education) and baseline clinical characteristics (BDI-II total score, past 30 days cocaine use). Separate multiple regressions were performed within cocaine and neutral trials using uncollapsed error rates to test differential associations within trial type. Third, regularized regression was utilized to model total error rates among the entire set of 21 individual BDI-II items. Regularization shrinks regression coefficients to prevent inflated estimates that may arise due to multicollinearity. Least absolute shrinkage and selection operator regression (LASSO, i.e., L1 regularization) permits variable selection by reducing small coefficients to zero, thus retaining only the most important predictors. The LASSO model provided penalized coefficients that may be interpreted in the same way as a traditional regression, relating the magnitude and direction of the association between the retained predictor and outcome variable.

No missingness was observed in the data. Normality assumptions for error rates and response times were confirmed graphically and numerically (skewness within ±1, kurtosis within ±3). All analyses were conducted on the R Statistical Computing Environment (v4.4.3; R Core Team, 2025) via the packages lme4 (GLMM) and glmnet (LASSO regression; Bates et al., 2015; Friedman et al., 2010). Variance explained by mixed models was estimated using semi-partial *R*^2^ (*R_m_*^2^) via the r2glmm package (Jaeger, 2017).

## Results

### Participant characteristics

A total of 101 participants completed eye-tracking measures at baseline and had available data for the current study, out of 118 participants randomized in the parent trial (Schmitz et al., 2024). Sample characteristics at baseline are presented in Table 1. Participants had a mean age of 49.5 (SD = 7.9, range 28–60) years, a mean education level of 12.9 years (SD = 1.5), and were mostly composed of male (81.2%), Black/African American (75.2%), non-Hispanic (88.1%) individuals. Participants had an average of 17.2 days of cocaine use in the past 30 days.

Less than half of participants had mild to severe depression (45.5%) on the BDI-II. More than half of participants reported above-zero levels in responses to the BDI-II items Loss of Pleasure (“I don’t enjoy things as much as I used to” to “I can’t get any pleasure from the things I used to enjoy”: 63.4%), Changes in Sleeping Pattern (“I sleep somewhat more than usual or I sleep somewhat less than usual” to “I sleep most of the day or I wake up 1–2 hours early and can’t back to sleep”: 63.4%), and Tiredness (“I get more tired or fatigued more easily than usual” to “I am too tired or fatigued to do most of the things I used to do”: 58.4%).

Pro-saccade error rates were 2.6% across blocks, indicating no gross pathology to retinal nerves, visual cortex, or frontal eye fields, and that participants were following task instructions. See Table S5 for mean response times and error rates on pro-saccade and anti-saccade trials. GLMM showed no significant effect of trial type on pro-saccade response times (*p* = 0.080, *R_m_*^2^ = 0.007) or error rates *p* = 0.271, *R_m_*^2^ = 0.002). No associations with BDI-II were found with pro-saccade response times or error rates (Table S6 and S7); and no BDI-II items were retained in the LASSO regression of pro-saccade error rates. Next, GLMM showed no significant effect of trial type on anti-saccade response times (*p* = 0.374, *R_m_*^2^ = 0.002). (See Tables S8–S10 for anti-saccade response times and cocaine-neutral error difference score results). Subsequent results focus on anti-saccade errors.

### Attentional bias

GLMM showed a significant effect on anti-saccade error rates, (*t*(100) = 4.76, *p* < 0.001, *d* = 0.67, *R_m_*^2^ = 0.061), with higher rates for cocaine (*M* = 0.49, SD = 0.25) than neutral trials (*M* = 0.38, SD = 0.24).

### Anti-saccade errors and baseline clinical variables

Multiple linear regression results of average error rates on demographic and baseline clinical characteristics are shown in Table 2. Analyses demonstrated a significant association with age (*p* = 0.040) and with BDI-II total score (*p* < 0.001) (*R*^2^_Adjusted_ = 0.202). Separate analyses within neutral trials (*R*^2^_Adjusted_ = 0.158) showed similar associations with age (*p* = .028) and BDI-II total score (*p* < 0.001); analyses within cocaine trials (*R*^2^_Adjusted_ = 0.141) showed significant associations with BDI-II total score only (*p* < 0.001).

**Table 2.** Multiple linear regression of anti-saccade error rate.

Variables	*B*	SE	*t*	*p*	*R* ^2^	Adj. *R*^2^
**Outcome: Average error rate**					0.266	0.202
(Intercept)	−0.176	0.225	−0.781	0.437		
BDI-II Total Score	0.009	0.002	5.079	**<0.001**		
Age	0.006	0.003	2.081	**0.040**		
Sex: Female (ref: Male)	−0.015	0.053	−0.285	0.776		
Race: White (ref: African American)	−0.095	0.063	−1.511	0.134		
Race: Other (ref: African American)	−0.174	0.104	−1.682	0.096		
Ethnicity: Hispanic (ref: Non-Hispanic)	0.161	0.096	1.681	0.096		
Education years	0.015	0.014	1.126	0.263		
Past 30 days cocaine use	0.001	0.002	0.276	0.783		
**Outcome: Cocaine trial error rate**					0.210	0.141
(Intercept)	−0.050	0.267	−0.187	0.852		
BDI-II Total Score	0.009	0.002	4.280	**<0.001**		
Age	0.004	0.003	1.316	0.191		
Sex: Female (ref: Male)	−0.049	0.063	−0.775	0.441		
Race: White (ref: African American)	−0.111	0.074	−1.488	0.140		
Race: Other (ref: African American)	−0.117	0.123	−0.956	0.342		
Ethnicity: Hispanic (ref: Non-Hispanic)	0.128	0.114	1.129	0.262		
Education years	0.014	0.016	0.847	0.399		
Past 30 days cocaine use	0.002	0.003	0.925	0.357		
**Outcome: Neutral trial error rate**					0.225	0.158
(Intercept)	−0.302	0.263	−1.149	0.253		
BDI-II Total Score	0.009	0.002	4.363	**<0.001**		
Age	0.007	0.003	2.233	**0.028**		
Sex: Female (ref: Male)	0.018	0.062	0.298	0.766		
Race: White (ref: African American)	−0.079	0.073	−1.080	0.283		
Race: Other (ref: African American)	−0.231	0.121	−1.913	0.059		
Ethnicity: Hispanic (ref: Non-Hispanic)	0.194	0.112	1.736	0.086		
Education years	0.017	0.016	1.071	0.287		
Past 30 days cocaine use	−0.001	0.003	−0.465	0.643		

Significant associations are in boldface (*p* < .05). Note: “Race: Other” collapsed across “Asian,” “More than one race,” and “Unknown/Not Reported” categories to permit sufficient *N* per category.

As a verification that the relationship with BDI-II total score did not differ by trial type, GLMM tested error rate as a function of the interaction between trial type and BDI-II total score, controlling for lower-order main effects, and found no significant interaction (*p* = 0.742, *R_m_*^2^ = 0.194; adjusting for age: *p* = 0.743, *R_m_*^2^ = 0.224).

Similar results as above were obtained when dichotomous BDI-II score (Mild or greater depression vs No depression) was used in lieu of the numeric total score (Supplemental Table S11).

### Anti-saccade errors and BDI-II items

Results from the LASSO regression of average error rates are shown in Table 3. Ten-fold cross-validation identified the optimized value for lambda (0.0219 given alpha = 1, the value for LASSO penalty). Of the 21 BDI-II items, seven were retained (listed from largest effect size): Agitation, Tiredness/Fatigue, Crying, Concentration Difficulty, Changes in Sleeping Pattern, Loss of Interest in Sex, and Loss of Pleasure. All regularized coefficients were positive, indicating higher severity in the retained items was associated with higher anti-saccade error rates. Coefficients ranged between 0.0004 (Loss of Pleasure) and 0.0456 (Agitation). Distributions of responses to the retained BDI-II items are shown in Table 1.

**Table 3.** LASSO regression of anti-saccade error rate.

BDI Item	Penalized coefficient	Cohen’s *d*
(Intercept)	0.3516	
(1) Sadness	NR	NR
(2) Pessimism	NR	NR
(3) Past Failure	NR	NR
(4) Loss of Pleasure	0.0004	0.0009
(5) Guilty Feelings	NR	NR
(6) Punishment Feelings	NR	NR
(7) Self-Dislike	NR	NR
(8) Self-Criticalness	NR	NR
(9) Suicidal Thoughts	NR	NR
(10) Crying	0.0305	0.0610
(11) Agitation	0.0456	0.0914
(12) Loss of Interest	NR	NR
(13) Indecisiveness	NR	NR
(14) Worthlessness	NR	NR
(15) Loss of Energy	NR	NR
(16) Changes in Sleeping Pattern	0.0020	0.0039
(17) Irritability	NR	NR
(18) Changes in Appetite	NR	NR
(19) Concentration Difficulty	0.0128	0.0256
(20) Tiredness/Fatigue	0.0363	0.0727
(21) Loss of Interest in Sex	0.0012	0.0024

NR = not retained.

## Discussion

The current study examined the relationship of anti-saccade error rates with picture type and with relevant baseline characteristics in participants seeking treatment for CUD. Results indicated higher anti-saccade error rates on cocaine trials, and significant associations of overall error rates with BDI-II total scores, controlling for demographic and baseline recent cocaine use variables. Error rates were most strongly related to seven BDI-II items, namely Agitation, Tiredness/Fatigue, Crying, Concentration Difficulty, Changes in Sleeping Pattern, Loss of Interest in Sex, and Loss of Pleasure. Findings were observed only on anti-saccade and not pro-saccade trials, supporting the hypothesis that the drug-specific bias and overall association with somatic depression symptoms in this population may be specific to response inhibition.

The drug specificity of error rates in the current study is consistent with attentional bias in CUD (Dias et al., 2015; Tannous et al., 2019) and across SUDs (Anderson, 2013; O’Neill et al., 2020; Roberts et al., 2014; Schröder and Mühlberger, 2022; Yoon et al., 2019). Drug stimuli acquire incentive salience over the course of repeated drug use, resulting in increased stimulus salience that captures attention in subsequent contexts (Robinson and Berridge, 1993). The current data confirm the drug stimulus-specificity of this bias (Marks et al., 2015).

Average anti-saccade error rates were positively associated with BDI-II total scores and age. Age effects are established in the literature and are unsurprising given the study sample represents an age interval (28–60 years) in which anti-saccade errors are expected to increase (Munoz et al., 1998). The relationship with BDI-II total score suggests that poor response inhibition is mainly driven by depressive symptoms in CUD. Contrary to our hypothesis, the association did not differ across cocaine and neutral trials, indicating that the association with depressive symptoms may reflect poor overall inhibitory control rather than drug-specific attention bias. Prior studies have shown generally worse inhibitory control and attentional processing in self-reported or diagnosed depression versus healthy controls across tasks (Ainsworth and Garner, 2013; Derakshan et al., 2009; Li et al., 2021). Of course, the present data are further complicated by the presence of CUD. Notably, both depression and CUD are related to abnormal dorsolateral prefrontal cortex (DLPFC) and anterior cingulate activation, with diminished cognitive control being linked to hypoactivity in these regions (Disner et al., 2011; Goldstein and Volkow, 2011; Koenigs et al., 2008; Pizzagalli, 2011; Sutherland et al., 2012).

The conclusion that depression was the main driver of response inhibition errors must be tempered by the observation that more than half (54.5%) of the current sample did not meet criteria for mild or severe depression, so the strength of this hypothesis should be tested in other CUD samples that have more severe depression. An alternative, and potentially complementary, explanation is that the observed inhibitory control deficits reflect physiological fatigue or sleep-related dysfunction associated with drug use, as suggested by the regularized regression on individual BDI-II items. Those analyses retained seven items, the majority of which (Agitation, Tiredness/Fatigue, Concentration Difficulty, Changes in Sleeping Pattern, Loss of Interest in Sex) aligned with the somatic dimension of the BDI-II established by prior factor analyses (Buckley et al., 2001; Seignourel et al., 2008). The somatic factor was previously shown to be elevated in stimulant-using populations, suggesting that the relationship between depressive symptoms and somatic symptomology arises from physiological effects of stimulant use (Johnson et al., 2006). Notably, the retained items in the current study revolve around tiredness/fatigue and sleep. Sleep deprivation is known to impair saccadic control (Zils et al., 2005) and alter attentional allocation (Woods et al., 2013), suggesting that sleep-and fatigue-related mechanisms may underlie the observed deficits in inhibitory control.

Notably, the relationship between somatic depressive symptoms and inhibitory control captures the physiological impacts of drug use as distinct from duration or severity of use, given there was no significant association with past 30-day cocaine use. This interpretation is consistent with neurobiological models linking sleep, fatigue, and prefrontal cortex function. Chronic stimulant use is associated with dysregulation of the DLPFC, a region critical for inhibitory control (Goldstein and Volkow, 2011; Sutherland et al., 2012). The DLPFC is also implicated in sleep-related fatigue and cognitive slowing (Soutschek and Tobler, 2020; Suda et al., 2009). Thus, the anti-saccade errors observed in this study may reflect a convergence of sleep-related and depression-related dysfunction in prefrontal systems. These findings raise the possibility that sleep disturbance and fatigue may be modifiable contributors to cognitive dysfunction in CUD. Interventions targeting sleep—such as cognitive behavioral therapy for insomnia—have shown promise in improving both mood and cognitive outcomes in depressed populations (Ng et al., 2015; Sweetman et al., 2021). Similarly, pharmacological agents known to improve sleep (e.g., the FDA-approved insomnia medication suvorexant) have been shown to improve inhibitory control and mood in rats (Gentile et al., 2018) and humans (Suchting et al., 2020; Webber et al., 2024). Finally, DLPFC stimulation, a well-known treatment for major depression, has also shown promise in improving cognitive function and response inhibition (Asgharian Asl and Vaghef, 2022; Corlier et al., 2020; Yu et al., 2022). Future research should examine whether improving sleep quality or depressive symptomology or both in CUD patients leads to measurable improvements in inhibitory control and attentional bias, potentially enhancing treatment outcomes.

We temper the findings with the study’s limitations. All reported measures were taken only at baseline, thus, temporal precedence between inhibitory control and depression-related somatic symptoms cannot be established. If cognitive enhancement is intended to be used as a therapeutic target in CUD and SUD, then its causal link to depression and substance use must be better defined. Next, associations with anti-saccades were limited to self-report measures. We raised the possibility of DLPFC function underlying the associations between the inhibitory control and depression factors related to somatic-affective symptomology. Future research might test this hypothesis within CUD populations by incorporating biobehavioral assays of attentional bias and inhibitory control including psychophysical and electrophysiological measures (e.g., event-related potentials), or biomarkers of inflammatory cytokines in tear fluid implicated in sleep disruption-related ocular dryness (Vavilina et al., 2023). Notably, commonly used direct measures of sleep (e.g., Insomnia Severity Index: Bastien, 2001; Pittsburgh Sleep Quality Index: Buysse et al., 1989) were not available for the majority of participants in this sample. Future studies should include direct measures of sleep to further understand how sleep-related variables may be key factors related to anti-saccade-BDI II associations. The participant sample was heterogeneous on age, gender, time since last drug use, and other substance use. Greater control of these factors would enable more precise predictions of anti-saccade error rates. Inclusion of different subgroups (e.g., veterans vs non-veterans, DiGirolamo et al., 2017) and a non-cocaine comparison group would permit examination of limiting conditions of the association between inhibitory control and depressive symptoms. Finally, the CUD sample in the current study was all treatment-seeking, and had predominantly subthreshold depression on average, hence, findings may not generalize to non-treatment-seeking CUD populations or those with more severe depression. The generalizability to other SUD populations is also unclear and must be tested in future research.

Results from this study provide insights into the relationship between inhibitory control and depression-related somatic symptomology in cocaine use. These findings warrant further investigation on neurocognitive mechanisms of executive functioning and possibly sleep impairment underlying substance use that may guide the development of cognitive targets for SUD treatment.

## Supplemental Material

sj-docx-1-jop-10.1177_02698811251399579 – Supplemental material for Anti-saccade error rates are associated with somatic depressive symptoms in cocaine use disorder Supplemental material, sj-docx-1-jop-10.1177_02698811251399579 for Anti-saccade error rates are associated with somatic depressive symptoms in cocaine use disorder by Constanza de Dios, Heather E. Webber, Margaret C. Wardle, Jin H. Yoon, Michelle A. Patriquin, Jessica N. Vincent, Joy M. Schmitz and Scott D. Lane in Journal of Psychopharmacology
