# Supplementary material for: Anti-saccade error rates are associated with somatic depressive symptoms in cocaine use disorder
Source: J Psychopharmacol. 2025 Dec 26;40(2):296–304. doi: 10.1177/02698811251399579 (PMC13222382; doi:10.1177/02698811251399579)
Supplement: sj-docx-1-jop-10.1177_02698811251399579 – Supplemental material for Anti-saccade error rates are associated with somatic depressive symptoms in cocaine use disorder [file sj-docx-1-jop-10.1177_02698811251399579.docx]

Anti-saccade error rates are associated with somatic depressive symptoms in cocaine use disorder

**Supplementary Materials**

# Supplementary Results

## Anti-saccade errors and baseline clinical variables

Multiple linear regression results of average error rates on demographic and baseline clinical characteristics are shown in Table S1. Analyses demonstrated a significant association with with BDI-II total score (*p*<.001). Separate analyses within neutral trials and within cocaine trials showed similar associations with BDI-II total score (*p*<.001). As a verification that the relationship with BDI-II dichotomous score did not differ by trial type, GLMM tested error rate as a function of the interaction between trial type and BDI-II total score, controlling for lower-order main effects, and found no significant interaction (*p*=0.527, *R_m_^2^*=0.190; adjusting for age: *p*=0.527, *R_m_^2^*=0.215).

No relationships with BDI-II total score or individual BDI-II items were found when cocaine-minus-neutral difference scores in error rates were used as the dependent measure (Tables S4-S6).

**Table S1**. Medications reported by participants at baseline.

| **Medications** | **Reasons** |
| --- | --- |
| Advil | Allergies |
| Aleve | Blood pressure/Hypertension |
| Alka seltzer | Cholesterol |
| Amlodipine | Digestion |
| Antibiotic | Flu/Respiratory |
| Aspirin | HIV |
| Cetirizine | Hyperthyroid |
| Claritin | Pain |
| Flexeril | Sleep |
| Flonase | Smoking |
| Gabapentin | Supplement |
| Hydrochlorothiazide | Unknown |
| Ibuprofen |  |
| Labetalol |  |
| Lisinopril |  |
| Losartan |  |
| Magnesium |  |
| Melatonin |  |
| Metoprolol |  |
| Milk thistle |  |
| Multivitamins |  |
| Nortriptyline |  |
| Norvir |  |
| Omega 3 |  |
| Omeprazole |  |
| Prezista |  |
| Seroquil |  |
| Simvastatin |  |
| Steroid |  |
| Tivicay |  |
| Tramadol |  |
| Truvada |  |
| Tylenol |  |
| Unknown |  |
| Valsartan |  |
| Wellbutrin |  |
| 5-Hydroxytryptophan |  |

**Table S2**. Anti-saccade response latencies in cocaine-dependent participants across studies that used eyetracking paradigm of the current study.

|  |  |  | **Response Latency (ms)** | |
| --- | --- | --- | --- | --- |
| **Study Keywords** | **Related publications (First Author, Year, PMID)** | **N CUD participants** | **M (SD)** | **Median (IQR)** |
| Cocaine Eye-tracking | Dias et al., 2015 (26164486) | 46 | 339 (68.7) | 344 (70.7) |
| Eyetracking and DTI baseline measures in comorbid CUD/AUD | Tannous et al., 2019 (30625144) | 24 | 311 (81) | 313 (108) |
| Orexin and sleep in CUD | Suchting et al., 2020 (31374218) | 21 | 346 (39.5) | 337 (45.8) |
| Adaptive Trial | Current study | 101 | 329 (56.9) | 331 (63.5) |
| Minimum |  |  | 311 | 313 |
| Maximum |  |  | 346 | 344 |
| Average |  |  | 331.3 | 331.25 |

Webber et al., 2022 (PMID 33630644) and de Dios et al. (PMID 33586502) were not included as these two studies used smaller subsets of the complete eyetracking sample of the current study.

**Figure S3**. Eyetracking trial sequence.


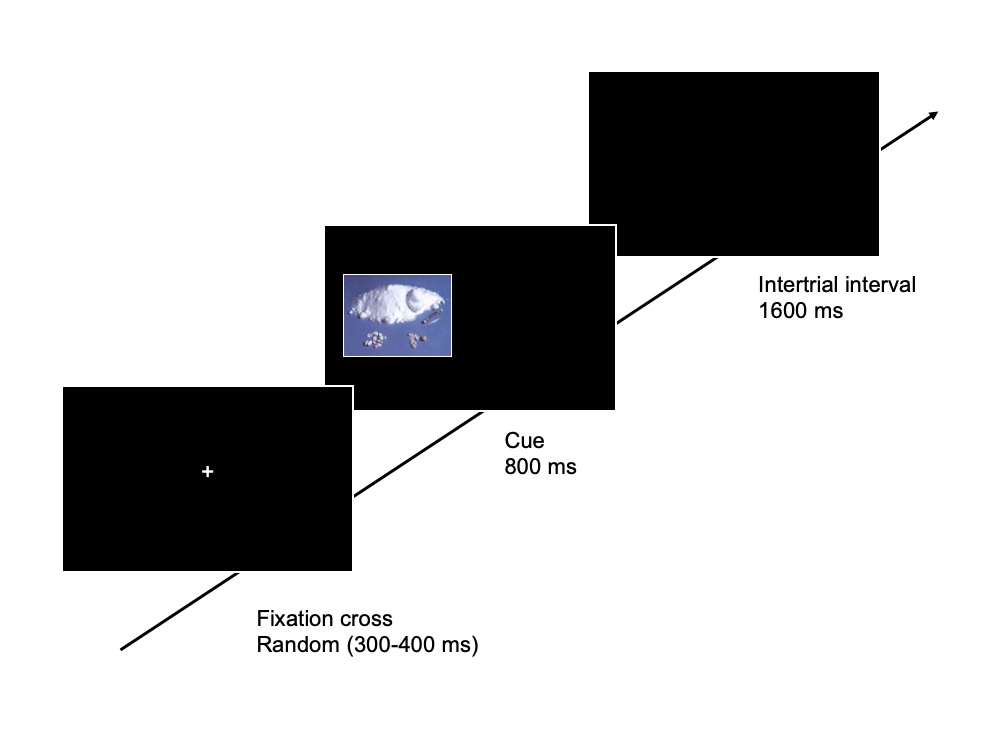


A central fixation crosshair signaled the start of a trial. The cue was one of six unique cocaine images or 6 unique neutral images, counterbalanced to the left or right visual hemifield. For pro-saccade trials, the participant was instructed to look at the image. Conversely, for the anti-saccade trials, the participant was instructed to look away from the image and to fixate on the blank screen on the opposite side. Four blocks were administered (2 pro-saccade, 2 anti-saccade) with 24 pro and 24 anti-saccade trials. Block order was fully counterbalanced across participants. Cocaine images were created from freely available online sources and matched as closely as possible to neutral images on visual characteristics such as color, background, complexity, and presence of people. Cocaine-related cues included powdered cocaine in isolation as well as close-ups of individuals’ hands as they smoked cocaine through pipes. Faces were not shown. Neutral cues included environmental scenes as well as close-ups of hands holding non-drug related objects. Each of the images (250 x 188 pixels) was presented on a 304 x 378 mm screen, either 7° to the left or right of the central fixation cross.

**Figure S4.** Example images used as cues in the eyetracking task.


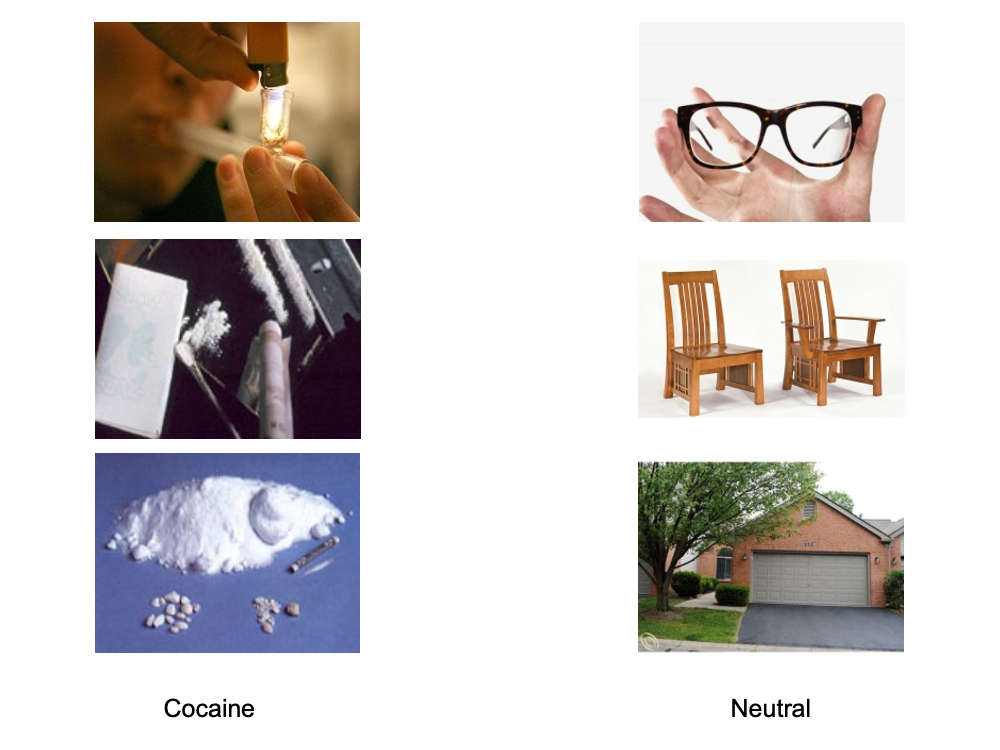


**Table S5.** Error rates and response times on anti-saccade and pro-saccade trials.

|  | All trials | | Cocaine trials | | Neutral trials | | |  |
| --- | --- | --- | --- | --- | --- | --- | --- | --- |
|  | M (SD) | Median (IQR) | M (SD) | Median (IQR) | | M (SD) | Median (IQR) | |
| **Error rate (%)** | | | | | | | |  |
| Pro-saccade trials | 2.6 (6.4) | 0 (2.1) | 2.3 (6.5) | 0 (4.2) | | 2.9 (6.8) | 0 (4.2) | |
| Anti-saccade trials | 43.6 (22.3) | 41.7 (31.3) | 49.4 (25.4) | 45.8 (37.5) | | 38.2 (25.3) | 37.5 (41.7) | |
| **Response time (ms)** | | | | | | | |  |
| Pro-saccade trials | 263 (44) | 270 (53) | 267 (47) | 272 (51) | | 260 (49) | 262 (50) | |
| Anti-saccade trials | 329 (53) | 333 (41) | 331 (57) | 332 (56) | | 328 (57) | 328 (65) | |

**Table S6.** Multiple linear regression results for pro-saccade error rate.

|  |  | *B* | *SE* | *t* | *p* | *R^2^* | *Adj. R^2^* |
| --- | --- | --- | --- | --- | --- | --- | --- |
|  | **Outcome: Average error rate** |  |  |  |  | 0.109 | 0.032 |
|  | (Intercept) | -5.178 | 1.024 | -5.059 | <0.001 |  |  |
|  | BDI-II Total Score | 0.012 | 0.008 | 1.529 | 0.130 |  |  |
|  | Age | 0.010 | 0.013 | 0.786 | 0.434 |  |  |
|  | Sex: Female (ref: Male) | -0.143 | 0.241 | -0.594 | 0.554 |  |  |
|  | Race: White (ref: African American) | -0.199 | 0.286 | -0.697 | 0.488 |  |  |
|  | Race: Other (ref: African American) | -0.808 | 0.472 | -1.713 | 0.090 |  |  |
|  | Ethnicity: Hispanic (ref: Non-Hispanic) | 0.969 | 0.436 | 2.220 | **0.029** |  |  |
|  | Education years | 0.029 | 0.062 | 0.471 | 0.639 |  |  |
|  | Past 30 days cocaine use | 0.015 | 0.010 | 1.464 | 0.147 |  |  |

Pro-saccade error rates were log-transformed prior to casting in the regression. Significant associations are in boldface (*p*<.05). Similar results were obtained when Dichotomous BDI-II score (Mild or greater depression vs. No depression) was used in place of the numeric BDI-II total score. Note: “Race: Other” collapsed across “Asian”, “More than one race”, and “Unknown/Not Reported” categories to permit sufficient N per category.

**Table S7**. Multiple linear regression results for pro-saccade response time (RT).

|  |  | *B* | *SE* | *t* | *p* | *R^2^* | *Adj. R^2^* |
| --- | --- | --- | --- | --- | --- | --- | --- |
|  | **Outcome: Average RT** |  |  |  |  | 0.108 | 0.031 |
|  | (Intercept) | 249.469 | 49.209 | 5.070 | <0.001 |  |  |
|  | BDI-II Total Score | -0.576 | 0.372 | -1.547 | 0.125 |  |  |
|  | Age | -0.125 | 0.612 | -0.204 | 0.839 |  |  |
|  | Sex: Female (ref: Male) | -23.856 | 11.572 | -2.062 | **0.042** |  |  |
|  | Race: White (ref: African American) | 6.802 | 13.736 | 0.495 | 0.622 |  |  |
|  | Race: Other (ref: African American) | -12.440 | 22.674 | -0.549 | 0.585 |  |  |
|  | Ethnicity: Hispanic (ref: Non-Hispanic) | -1.368 | 20.973 | -0.065 | 0.948 |  |  |
|  | Education years | 3.178 | 2.968 | 1.071 | 0.287 |  |  |
|  | Past 30 days cocaine use | -0.415 | 0.483 | -0.858 | 0.393 |  |  |

Significant associations are in boldface (*p*<.05). Similar results were obtained when Dichotomous BDI-II score (Mild or greater depression vs. No depression) was used in place of the numeric BDI-II total score. Note: “Race: Other” collapsed across “Asian”, “More than one race”, and “Unknown/Not Reported” categories to permit sufficient N per category.

**Table S8**. Multiple linear regression results for anti-saccade response time (RT).

|  |  | *B* | *SE* | *t* | *p* | *R^2^* | *Adj. R^2^* |
| --- | --- | --- | --- | --- | --- | --- | --- |
|  | **Outcome: Average RT** |  |  |  |  | 0.075 | -0.005 |
|  | (Intercept) | 438.841 | 59.775 | 7.342 | <0.001 |  |  |
|  | BDI-II Total Score | -0.247 | 0.452 | -0.546 | 0.587 |  |  |
|  | Age | -1.743 | 0.744 | -2.344 | **0.021** |  |  |
|  | Sex: Female (ref: Male) | -11.510 | 14.057 | -0.819 | 0.415 |  |  |
|  | Race: White (ref: African American) | 20.860 | 16.686 | 1.250 | 0.214 |  |  |
|  | Race: Other (ref: African American) | -5.395 | 27.543 | -0.196 | 0.845 |  |  |
|  | Ethnicity: Hispanic (ref: Non-Hispanic) | -8.808 | 25.476 | -0.346 | 0.730 |  |  |
|  | Education years | -1.504 | 3.605 | -0.417 | 0.678 |  |  |
|  | Past 30 days cocaine use | 0.023 | 0.587 | 0.039 | 0.969 |  |  |

Significant associations are in boldface (*p*<.05). Similar results were obtained when Dichotomous BDI-II score (Mild or greater depression vs. No depression) was used in place of the numeric BDI-II total score. Note: “Race: Other” collapsed across “Asian”, “More than one race”, and “Unknown/Not Reported” categories to permit sufficient N per category.

**Table S9.** Multiple linear regression results for anti-saccade error rate difference score (cocaine - neutral)

|  | *B* | *SE* | *t* | *p* | *R^2^* | *Adj. R^2^* |
| --- | --- | --- | --- | --- | --- | --- |
| **Outcome: Error rate difference score** |  |  |  |  | 0.054 | -0.028 |
| (Intercept) | 0.252 | 0.278 | 0.906 | 0.367 |  |  |
| BDI-II Total Score | -0.000 | 0.002 | -0.016 | 0.987 |  |  |
| Age | -0.003 | 0.003 | -0.847 | 0.399 |  |  |
| Sex: Female (ref: Male) | -0.067 | 0.065 | -1.024 | 0.308 |  |  |
| Race: White (ref: African American) | -0.032 | 0.078 | -0.407 | 0.685 |  |  |
| Race: Other (ref: African American) | 0.114 | 0.128 | 0.889 | 0.376 |  |  |
| Ethnicity: Hispanic (ref: Non-Hispanic) | -0.066 | 0.119 | -0.557 | 0.579 |  |  |
| Education years | -0.003 | 0.017 | -0.199 | 0.843 |  |  |
| Past 30 days cocaine use | 0.004 | 0.003 | 1.326 | 0.188 |  |  |

Significant associations are in boldface (*p*<.05). Similar results were obtained when Dichotomous BDI-II score (Mild or greater depression vs. No depression) was used in place of the numeric BDI-II total score. Note: “Race: Other” collapsed across “Asian”, “More than one race”, and “Unknown/Not Reported” categories to permit sufficient N per category.

**Table S10.** LASSO regression results (Outcome: Cocaine – Neutral cue error rate difference score).

| **BDI Item** | **Penalized coefficient** |
| --- | --- |
| (Intercept) | 0.1118 |
| (1) Sadness | NR |
| (2) Pessimism | NR |
| (3) Past Failure | NR |
| (4) Loss of Pleasure | NR |
| (5) Guilty Feelings | NR |
| (6) Punishment Feelings | NR |
| (7) Self-dislike | NR |
| (8) Self-criticalness | NR |
| (9) Suicidal Thoughts | NR |
| (10) Crying | NR |
| (11) Agitation | NR |
| (12) Loss of Interest | NR |
| (13) Indecisiveness | NR |
| (14) Worthlessness | NR |
| (15) Loss of Energy | NR |
| (16) Changes in Sleeping Pattern | NR |
| (17) Irritability | NR |
| (18) Changes in Appetite | NR |
| (19) Concentration Difficulty | NR |
| (20) Tiredness/Fatigue | NR |
| (21) Loss of Interest in Sex | NR |

NR = not retained.

**Table S11**. Multiple linear regression results for anti-saccade error rate.

|  |  | *B* | *SE* | *t* | *p* | *R^2^* | *Adj. R^2^* |
| --- | --- | --- | --- | --- | --- | --- | --- |
|  | **Outcome: Average error rate** |  |  |  |  | 0.243 | 0.177 |
|  | (Intercept) | -0.031 | 0.226 | -0.136 | 0.892 |  |  |
|  | BDI-II: Mild or greater depression (ref: No depression) | 0.199 | 0.042 | 4.711 | **<0.001** |  |  |
|  | Age | 0.005 | 0.003 | 1.847 | 0.068 |  |  |
|  | Sex: Female (ref: Male) | -0.020 | 0.054 | -0.365 | 0.716 |  |  |
|  | Race: White (ref: African American) | -0.069 | 0.063 | -1.085 | 0.281 |  |  |
|  | Race: Other (ref: African American) | -0.167 | 0.105 | -1.589 | 0.115 |  |  |
|  | Ethnicity: Hispanic (ref: Non-Hispanic) | 0.171 | 0.098 | 1.745 | 0.084 |  |  |
|  | Education years | 0.008 | 0.014 | 0.581 | 0.563 |  |  |
|  | Past 30 days cocaine use | 0.001 | 0.002 | 0.662 | 0.510 |  |  |
|  | **Outcome: Cocaine trial error rate** |  |  |  |  | 0.208 | 0.139 |
|  | (Intercept) | 0.092 | 0.263 | 0.351 | 0.727 |  |  |
|  | BDI-II: Mild or greater depression (ref: No depression) | 0.209 | 0.049 | 4.241 | **<0.001** |  |  |
|  | Age | 0.004 | 0.003 | 1.175 | 0.243 |  |  |
|  | Sex: Female (ref: Male) | -0.054 | 0.063 | -0.859 | 0.393 |  |  |
|  | Race: White (ref: African American) | -0.085 | 0.074 | -1.151 | 0.253 |  |  |
|  | Race: Other (ref: African American) | -0.113 | 0.123 | -0.923 | 0.359 |  |  |
|  | Ethnicity: Hispanic (ref: Non-Hispanic) | 0.140 | 0.114 | 1.230 | 0.222 |  |  |
|  | Education years | 0.006 | 0.016 | 0.361 | 0.719 |  |  |
|  | Past 30 days cocaine use | 0.003 | 0.003 | 1.246 | 0.216 |  |  |
|  | **Outcome: Neutral trial error rate** |  |  |  |  | 0.192 | 0.122 |
|  | (Intercept) | -0.154 | 0.264 | -0.582 | 0.562 |  |  |
|  | BDI-II: Mild or greater depression (ref: No depression) | 0.189 | 0.049 | 3.811 | **<0.001** |  |  |
|  | Age | 0.007 | 0.003 | 1.980 | 0.051 |  |  |
|  | Sex: Female (ref: Male) | 0.015 | 0.063 | 0.233 | 0.816 |  |  |
|  | Race: White (ref: African American) | -0.052 | 0.074 | -0.705 | 0.483 |  |  |
|  | Race: Other (ref: African American) | -0.221 | 0.123 | -1.792 | 0.076 |  |  |
|  | Ethnicity: Hispanic (ref: Non-Hispanic) | 0.201 | 0.115 | 1.753 | 0.083 |  |  |
|  | Education years | 0.010 | 0.016 | 0.631 | 0.529 |  |  |
|  | Past 30 days cocaine use | 0.000 | 0.003 | -0.113 | 0.911 |  |  |

Dichotomous BDI-II score (Mild or greater depression vs. No depression) was used in place of the numeric BDI-II total score. Significant associations are in boldface (*p*<.05). Note: “Race: Other” collapsed across “Asian”, “More than one race”, and “Unknown/Not Reported” categories to permit sufficient N per category.

**References**

Dias, N. R., Schmitz, J. M., Rathnayaka, N., Red, S. D., Sereno, A. B., Moeller, F. G., & Lane, S. D. (2015). Anti-saccade error rates as a measure of attentional bias in cocaine dependent subjects. *Behavioural Brain Research*, *292*, 493–499. https://doi.org/10.1016/j.bbr.2015.07.006

de Dios, C., Suchting, R., Webber, H. E., Yoon, J. H., Yammine, L., Vincent, J., Weaver, M. F., Stotts, A. L., Schmitz, J. M., & Lane, S. D. (2021). Cocaine-specific speed-accuracy trade-off during anti-saccade testing differentiates patients with cocaine use disorder who achieve initial abstinence during treatment. *Journal of Psychopharmacology*, *35*(5), 611–614. https://doi.org/10.1177/0269881121991566

Suchting, R., Yoon, J. H., Miguel, G. G. S., Green, C. E., Weaver, M. F., Vincent, J. N., Fries, G. R., Schmitz, J. M., & Lane, S. D. (2020). Preliminary examination of the orexin system on relapse-related factors in cocaine use disorder. *Brain Research*, *1731*(July 2019), 146359. https://doi.org/10.1016/j.brainres.2019.146359

Tannous, J., Mwangi, B., Hasan, K. M., Narayana, P. A., Steinberg, J. L., Walss-Bass, C., Gerard Moeller, F., Schmitz, J. M., & Lane, S. D. (2019). Measures of possible allostatic load in comorbid cocaine and alcohol use disorder: Brain white matter integrity, telomere length, and anti-saccade performance. *PLoS ONE*, *14*(1), 1–17. https://doi.org/10.1371/journal.pone.0199729

Webber, H. E., de Dios, C., Wardle, M. C., Suchting, R., Green, C. E., Schmitz, J. M., Lane, S. D., & Versace, F. (2021). Electrophysiological responses to emotional and cocaine cues reveal individual neuroaffective profiles in cocaine users. *Experimental and Clinical Psychopharmacology*. https://doi.org/10.1037/pha0000450
